# Exposure to Stress and Burnout Syndrome in Healthcare Workers, Expert Workers, Professional Associates, and Associates in Social Service Institutions

**DOI:** 10.3390/medicina60030499

**Published:** 2024-03-19

**Authors:** Snežana Marković, Olivera Kostić, Zorica Terzić-Supic, Sanja Tomic Mihajlovic, Jasmina Milovanović, Snezana Radovanovic, Nebojša Zdravković, Vladislava Stojić, Ljiljana Jovčić, Biljana Jocić-Pivač, Aleksandra Tomić Lučić, Marina Kostić, Marija Šorak

**Affiliations:** 1 Faculty of Medical Sciences, University of Kragujevac, 34000 Kragujevac, Serbia; snezana.markovic@czodo.rs (S.M.); tmsanja@gmail.com (S.T.M.); 2 Department of Pharmacy, Faculty of Medical Sciences, University of Kragujevac, 34000 Kragujevac, Serbia; olivera.milovanovic09@gmail.com; 3 Center for Research on Harmful Effects of Biological and Chemical Hazards, Faculty of Medical Sciences, University of Kragujevac, 34000 Kragujevac, Serbia; jovanarad@yahoo.com (S.R.); nzdravkovic@gmail.com (N.Z.); vladislavastojic@gmail.com (V.S.); marrina2006kg@yahoo.com (M.K.); 4 Institute of Social Medicine, Serbia Faculty of Medicine, University of Belgrade, 11000 Belgrade, Serbia; zorica.terzic-supic@med.bg.ac.rs; 5 Department of Pharmacology and Toxicology, Faculty of Medical Sciences, University of Kragujevac, 34000 Kragujevac, Serbia; jasminamilo@yahoo.com; 6 Department of Social Medicine, Faculty of Medical Sciences, Universitz of Kragujevac, 34000 Kragujevac, Serbia; 7 Department of Biomedical Statistics and Informatics, Faculty of Medical Sciences, University of Kragujevac, 34000 Kragujevac, Serbia; 8 Academy of Professional Studies Belgrade, Department of the Higher School of Health, Department of Health Care, 11000 Belgrade, Serbia; ljiljana.jovcic@yahoo.com; 9 Medical Faculty, University of Belgrade, 11000 Belgrade, Serbia; bjocicpivac@hotmail.com; 10 Department of Internal Medicine, Faculty of Medical Sciences, University of Kragujevac, 34000 Kragujevac, Serbia; atomiclucic@gmail.com; 11 Clinic for Rheumatology and Allergology, University Clinical Center of Kragujevac, 34000 Kragujevac, Serbia; 12 Department of Gynecology and Obstetrics, Faculty of Medical Sciences, University of Kragujevac, 34000 Kragujevac, Serbia; 13 Department of Biomedically Assisted Fertilization, Clinic of Gynecology and Obstetrics, University Clinical Center Kragujevac, 34000 Kragujevac, Serbia

**Keywords:** burnout syndrome, stress, resilience, social institutions, job-professional person

## Abstract

*Background and Objectives:* Workplace burnout syndrome is often as sociated with particular aspects of certain job positions, especially those that entail working with people with special needs. The burnout syndrome in healthcare jobs is a serious problem that has grown into an epidemic among healthcare workers and associates. The aim of this research is to assess the presence of stress and burnout syndrome at work with healthcare workers, expert workers, professional associates, and associates in social service institutions in Belgrade. *Materials and Methods*: This research was conducted in the form of a cross-sectional study of a representative sample in social institutions in Belgrade. It was conducted from March to the end of June of 2023. The sample of the study had 491 participants. The questionnaires used were a structured instrument with social–demographic and social–economic characteristics, workplace characteristics, lifestyle characteristics, and the following questionnaires: DASS-21, Copenhagen, Brief Resilience Scale, and Brief Resilient Coping Scale. *Results*: The end results indicate the following to be significant risk factors for the occurrence of workplace burnout syndrome: overtime (OR = 2.62; CI = 1.50–4.56), BRS average score (OR = 0.28; CI = 0.17–0.44), DASS21 D heightened depression (OR = 2.09; CI = 1.1–4.04), DASS21 A heightened anxiety (OR = 2.38; CI = 1.34–4.21), and DASS21 S heightened stress (OR = 2.08; CI = 1.11–3.89). The only protective risk factor that stood out was the self-assessment of health levels (OR = 0.60; CI = 0.42–0.85). *Conclusion:* Overtime is a significant factor associated with workplace burnout. Apart from it, other significant factors associated with workplace burnout were heightened depression, anxiety, and stress levels.

## 1. Introduction

In today’s modern world, stress has become an inevitable part of human life. Its causes and effects are numerous, and many individuals are continuously exposed to stress amid difficult professional circumstances. Stress, especially for those working in healthcare and social services, can have a deep and significant impact on the physical, mental, and emotional state of an individual.

Today, different behavior patterns and illnesses are related to stress occurrence. The notion of stress can be found in the literature as the notion of many scientific disciplines. Still, as it is inevitably tied to it, it is mostly connected to jobs where communication and working with people are predominant [1]. Workload significantly impacts exhaustion, work quality, motivation, and job satisfaction. All of these are conditions that interfere with team communication, cause fatigue, and together they endanger the health of the employees as well as patients’ safety [2]. Extended exposure to situations that the organism recognizes as a threat may lead to a number of disorders that all have a negative impact on basic life functions and the working abilities of a person, as well as their health state [3]. Inadequate communication and especially bad relationships are factors that add to the development of this syndrome [4]. The burnout syndrome occurs after long-term exposure to significant stress, especially in situations of others’ great expectations. In the literature, burnout is defined as a state of emotional exhaustion, depersonalization, and reduced personal achievement and accomplishment [5]. By another definition, burnout syndrome is “a state of mental and/or physical exhaustion caused by excessive and prolonged stress” [6]. According to the data so far, this is classified as professional stress and has an impact on job positions in regard to working with people in job spheres predominantly oriented to providing certain help to other people. Research with people of different job profiles showed higher tendencies towards burnout with people of great potential and great expectations, i.e., ambition, and the riskiest groups are the healthcare, social services, and education work fields [7].

Out of 40 participating job positions, the highest stress levels were found in healthcare workers, as published by the American National Association of Safety Professionals. In their study, they concluded that medical care workers are significantly more exposed to stress, and, unlike many other jobs, they show organism exhaustion, absence from work, environment, and family problems, as well as mental and physical disorders, according to Engen Maye et al. in 2013 [8].

Within their profession, social workers face numerous difficulties while working with different profiles, including persons with psychological problems, developmental problems, and dementia. Once the workload of the social worker becomes too heavy, and the personal well-being is constantly put aside, the burnout risk grows significantly. The burnout may be worsened by feeling tired and vice versa, which is the essential reason social workers and associates recognize and understand the signs and symptoms of both [1]. Workplace stress was studied as well, and it was pointed out that work management, career advancement, the role of the individual, work tasks, work environment, work conditions, and working in shifts are the most significant groups of stressors. Long-term exposure to those stressors may lead to burnout syndrome characterized by mental, physical, or both mental and physical exhaustion [5].

Workplace burnout syndrome is often associated with particular aspects of certain jobs, especially those in relation to people with difficulties. According to the research of workplace burnout syndrome, jobs such as medical and social workers, prison workers, and lawyers are particularly sensitive [5].

Socio-demographic variables are among the personal factors associated with burnout in the workplace. Thus far, factors such as age, education, marital status, and work experience have been identified in the literature as correlating with burnout syndrome. However, further research is necessary to better understand this correlation [5,7].

Burnout syndrome in healthcare is a serious problem, an epidemic among healthcare workers and associates. A damaging impact on the psycho-physical health of the employees has been confirmed, as well as the quality of services they provide, patients’ safety, and the maintenance of the health system [9]. The prevalence of burnout syndrome among healthcare workers is varied, yet the majority of studies show it to be around 50% [10].

Burnout syndrome may also have a long-term impact on the mental health of the healthcare workers, as well as the quality of their lives. Its prevention demands an active management of resources and changes in the work environment, as well as the development of personal characteristics that can help with stress.

The main aim of this research is to evaluate the presence of stress and burnout syndrome in healthcare workers, expert workers, professional associates, and associates in social protection institutions, and to examine the impact of certain social–demographic and work environment characteristics on the occurrence of burnout syndrome on the population in question.

## 2. Materials and Methods

### 2.1. Study Design

This research was designed as a cross-sectional study and conducted from March to the end of June of 2023 on a representative sample in social institutions in Belgrade. The total research sample consisted of 491 respondents, namely 204 (41.55%) health workers, 154 (31.36%) professional workers, 41 (8.35%) professional associates, and 92 (18.74%) associates in direct work with the users of social protection institutions on residence at the following institutions in Belgrade, Serbia:

1. Babies, children, and the youth protection center in Belgrade;
2. Adults and the elderly residence institution in Belgrade;
3. Developmentally challenged children and the youth daycare center in Belgrade;
4. Children’s and the youth’s institution in Sremčica.

The total number of employees was 774 in all four institutions, of which 502 (64.86%) employees participated in our research. However, due to missing data, 11 questionnaires were excluded from the research, so the number of respondents was 491 with a response rate of 63.44%. The common criteria for all participants were as follows: adults, both genders, older than 21, certain education profile, work experience over three years, and volunteering in the study. The excluding factors were persons younger than 21, work experience shorter than three years, fixed-term contracts, diagnosed mental disorders, and longer work absences (6 months) prior to the research. The participants, after giving consent, completed questionnaires with the explanations given by the main researcher and the adequate help given by the researchers, if needed.

### 2.2. Research Instruments

The structured research instrument had the questions regarding social–demographic and social–economic characteristics, work environment characteristics, and lifestyle characteristics. For the workplace burnout assessment, Copenhagen Burnout Inventory, CBI, was used. It has 19 questions dealing with exhaustion and tiredness relating to the burnout in the following aspects: personal burnout (questions 1–6), work-related burnout (questions 7–13), and client-related burnout (questions 14–19) [11]. The validity and reliability of this questionnaire have been confirmed through previous research. Cronbach’s alpha was higher than 0.7 for each domain of the questionnaire, as well as for the questionnaire in total, while the composite reliability values for the three factors varied between 0.84 and 0.87 [11,12].

The participants had the task of choosing the option closest to how they feel, following the frequency on the 5-level Likert scale in the span 0–4. The scoring was conducted by transforming the answers to time percentages: 0 = 0%, 1 = 25%, 2 = 50%, 3 = 75%, and 4 = 100%, according to the instructions by the questionnaire’s author. The score on each scale was calculated as the average score on the questions the scale entails, and the total score of workplace burnout was calculated as the average score of all three scales together, i.e., the average value of the separate scales’ scores. All the participants with scores over 50% are considered to have workplace burnout syndrome.

For the employees’ resilience examination, the Brief Resilience Scale (BRS) was used, created by Smith et al. [13]. This scale assesses the resilience construct, seen as the individual’s ability to bear with the problems in their environment and recover from stressful situations. The BRS is a one-dimensional scale that has 6 items. The total score on this scale is the arithmetic mean of all six.

Apart from BRS, the Brief Resilience Coping Scale (BRCS) was used. It has 4 items.

Stress, depression, and anxiety were assessed through the DASS-21 questionnaire. The DASS-21 has 21 items and includes three sub-scales with seven items. The participants had to assess their feelings from the past week on the Likert 4-span scale, and following the frequency on the Likert 5-span scale [14]. Depression, anxiety, and stress scores are the sum of the relevant scores in the 0–21 span for each sub-scale. The seriousness of the symptoms was calculated by cut-off scores for defining normal, mild, moderate, significant, and very significant scores for all sub-scales. 

For the “D” scale, a total score of 0–4 is normal; 5–6 mild depression; 7–10 moderate depression; 11–13 severe depression; ≥14 very severe depression. For the “A” scale, the score of 0–3 is considered normal; 4–5 mild anxiety; 6–7 moderate anxiety; 8–9 severe anxiety; ≥10 very severe anxiety. For the “C” scale, the score of 0–7 is normal; 8–9 mild stress; 10–12 moderate stress; 13–16 severe stress; ≥17 very severe stress.

### 2.3. Ethical Aspects of the Research

The research was conducted following the approval for it from the Ethical boards of the social service institutions and with consent of the participants. The ethical standards of research are aligned with the international (Helsinki Declaration) and specific legislation of our country. In order to respect the privacy of the research subjects and the confidentiality of information, all necessary steps were taken in accordance with the Law on Personal Data Protection (“Official Gazette of the Republic of Serbia”, No. 97/08, 104/09), the Law on Official Statistics (“Official Gazette of the Republic of Serbia”, No. 104/09), and the Directive of the European Parliament on personal data protection (Directive 95/46/EC). Informed consent was obtained from all subjects included in this study. The respondents’ consent form contained information about the purpose and method of conducting the research, as well as the method of data use.

### 2.4. Statistical Data Processing

For the primary data processing, descriptive statistical methods, methods for testing statistical hypotheses, and methods for modeling relations of the outcomes and potential predictors were used. Depending on variable types and normality of distribution, the data description was shown as N(%), arithmetic mean ± standard deviation (C), or median (min–max). Regarding methods for testing statistical hypotheses, tests used were the *t*-test, Mann–Whitney test, chi-square test, Fisher test, ANOVA, and Kruskal–Wallis test. For the modeling of relations of dependent variables with potential predictors, logistic regression was used. The multivariate regression models included predictors from uni-variant analyses that were statistically significant at the level of 0.05. For the internal consistency of the questionnaire assessment, the Cronbach’s alpha was used. Statistical hypotheses were tested based on the level of statistical significance (alpha level) of 0.05. The results were shown in tables and graphically. All data were processed in IBM SPSS Statistics 22 (SPSS Inc., Chicago, IL, USA) software package, R program environment (R version 4.2.3), (R Core Team, 2022).

## 3. Results

This research had a total of 491 participants, 60 (12.2%) men and 431 (87.8%) women. The average age was 45.9 years (95% CI 45.0–46.8). The youngest person was 21 and the oldest was 64. The arithmetic mean of the Copenhagen workplace burnout questionnaire research was 55.4 (95% CI 53.8–57.1). The lowest result was 3.9 and the highest was 100.0. The highest average score was on the question SPD1—*Do you consider work in social service institution to be hard?* (70.8%), and the lowest was on LS6—*How often do you feel weak and prone to illness?* (41.4%). The skewness and kurtosis values show that there was no deviation from the normal distribution in the questionnaire (Table 1).

**Figure 1:**
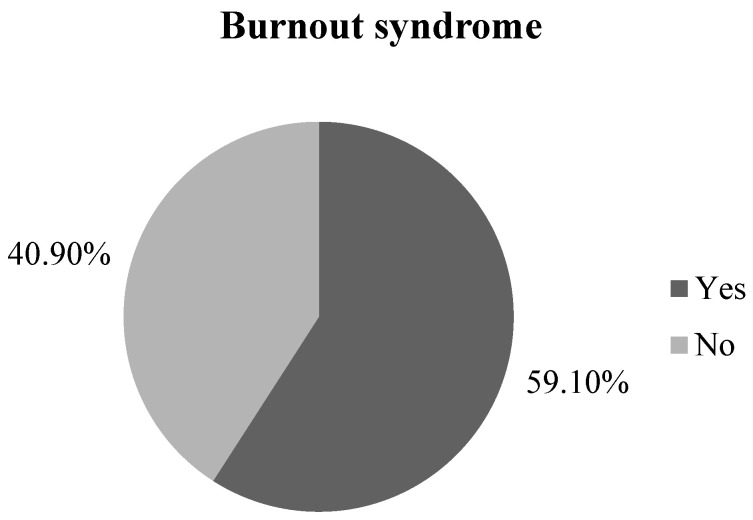
The distribution of participants according to the presence of workplace burnout.

The distribution of participants according to the presence of workplace burnout is shown in Figure 1.

There is a statistically significant difference between participants with and without workplace burnout syndrome in relation to gender, age, education, presence of children in the family, and the self-assessment of monthly income (Table 2).

There is a statistically significant difference between the participants with and without workplace burnout syndrome in relation to the characteristics of the work environment: occupation, overtime, shift work, adequate resources, and work in spacious and pleasant rooms (Table 3).

There is a statistically significant difference in participants with and without workplace burnout syndrome in relation to smoking habits, number of cigarettes, using sick leave, amount of sick leave, and self-assessed health (Table 4).

There is a statistically significant difference between participants with and without WBS in relation to the assessment of depression, anxiety, and stress according to the DASS-21 scale, and resilience according to the BRCS scale; the statistically significant differences are in relation to the degree of DASS-21 D, degree of DASS-21 A, degree of DASS-21 S, degree of BRCS, and the average score on BRS (Table 5).

After applying univariate analysis, 15 variables were included in the multivariate regression model (Table 6). The entire model, with all predictors, was statistically significant (*p* < 0.001). There is no significant multicollinearity among the predictors. The model describes a 51% variation of the dependent variable. In the model of multivariate logistic regression, the statistically significant WBS factors were overtime (OR = 2.62; CI = 1.50–4.56), BRS average score (OR = 0.28; CI = 0.17–0.44), DASS-21 D heightened depression (OR = 2.09; CI = 1.1–4.04), DASS-21 A heightened anxiety (OR = 2.38; CI = 1.34–4.21), and DASS-21 S heightened stress (OR = 2.08; CI = 1.11–3.89). The only protective risk factor that stands out is self-assessed health (OR = 0.60; CI = 0.42–0.85), i.e., the higher self-assessed health levels the participants had, the lower their chance for WBS was.

## 4. Discussion

The aim of this research was to determine the presence of stress and burnout syndrome in healthcare workers, expert workers, professional associates, and associates working in social service institutions. The aim was also to study the impact of certain social–demographic characteristics, as well as workplace characteristics, on the presence of burnout syndrome in the participating population. Doctors, nurses, counselors, and social workers often face trauma and others’ suffering, which is fertile ground for indirect trauma, often referred to as “empathy fatigue”. Providing others with help and support may in time lead to exhaustion and lowered ability for efficient work, which can in turn evolve into burnout syndrome [5]. So far, contributing factors have been identified for workplace burnout syndrome in people working in children’s protection and well-being, those who have endured excessive burden and demands of work with little to no control of it, those who have endured violence threats, and also those working with trauma victims and victims of stressful life events [15]. The generalization of the studies’ conclusions, which questioned the predictors of workplace burnout syndrome in the institutions for children’s protection and well-being, is limited by the differences in working conditions and work management in different countries [16].

The mean score on the CBI scale in our study was 55.4, which was higher than the scores observed in other surveyed populations in Serbia. Specifically, medical students had a mean score of 42.27, while preschool teachers had a mean score of 39.1 [17,18]. In this study, out of all the participants, over half of them showed signs of workplace burnout syndrome, i.e., 59.1%, while the lower part, 40.9%, showed no WBS development. The results of a North Carolina study show 39% of workplace burnout syndrome symptoms in social workers, which is quite similar in percentages to this study [19].

Female participants in this study showed a higher percentage of WBS than male participants. As one of the domains of this syndrome is emotional exhaustion, it can be seen that gender is a significant variable and that women experience higher levels of burnout, which can be explained by the societal role of women, but also by their constant tendencies to establish the balance between professional and private lives [20]. A study conducted among female resident physicians discovered that professional coaching led to reductions in emotional exhaustion and burnout scores, while also resulting in increased self-compassion scores. These findings suggest that such interventions may also be successful in addressing burnout in other professions where this syndrome is prevalent [21].

When it comes to the participants’ age, the results of this study have shown that those with workplace burnout syndrome were, on average, older than those without. Contrary to those results, it was earlier shown that burnout is more common in younger people, which can be explained by excessive enthusiasm and unrealistic workplace expectations, which are unsatisfactory in reality [22]. Age has been often mentioned in the literature as one of the individual workplace burnout syndrome factors, with the tendency to appear in younger employees [23].

In this study, the highest percentage of participants who have developed workplace burnout syndrome said they are not smokers. The results of another study, however, have shown that tobacco use enhances the risk of this syndrome [24], which this study does not show. These results may be explained by the fact that tobacco use is a stress management mechanism, a pleasure, and an escape from stressful work activities, which shows relaxing and anxiolytic effects [25].

Apart from working hours, professional status and professional titles were also closely tied to burnout syndrome. Out of all the participants in this study, the largest number had a high school level of education. When it comes to academic titles, a study conducted in Turkey implied that the amount of burnout decreases with higher levels of academic titles [26]. The results of this study were consistent with the results of the study of workplace burnout syndrome, and the joint factors in relation to healthcare workers, where it was shown that those with the lowest education were 1.57 times more prone to emotional exhaustion in relation to those with higher education [27].

One of the results of this research was that the persons with developed WBS had more years of experience in the field. When it comes to the levels of burnout with employees of children’s protection and well-being institutions, those who have more than a year of experience showed higher levels of burnout, which is in line with these research results [28].

All the previously listed factors that showed an impact in the univariate analysis did not remain significant in the multivariate model. A significant factor that did stand out in the latter was overtime, i.e., the participants who had overtime showed a 2.6 times higher risk of WBS compared to those with no overtime, with control of all other factors in the multivariate analysis model. So far, the literature has shown the connection between short-term and long-term working hours and burnout syndrome among healthcare workers. Healthcare workers with over 60 h weekly were twice as prone to WBS compared to a standard 40 h week. The authors of that study pointed out the linear rise of the chances for burnout with the larger number of hours, to 74 per work week (three times more likely) and 84 h per week (four times as likely) [29]. Taking this into account, the identification of overtime as a significant factor associated with burnout suggests the need for interventions aimed at reducing workload or implementing effective time management strategies in the workplace.

In general, mental health problems in healthcare workers show a high degree of coexistence, which can be explained by their exposure to the patients’ illnesses and a stressful workplace environment. Thus, they are inevitably more vulnerable to showing stress, anxiety, and depression [29,30,31,32]. Even though the highest percentage of participants (over 50%) had normal depression, anxiety, and stress levels, this research shows that, when they are heightened, they are seen as workplace burnout syndrome predictors. Persons with heightened depression, anxiety, and stress are more likely to experience WBS—2, 2.4, and 2.1 times, respectively—with other factors in the multivariate analysis controlled. It was previously shown that depression and stress are statistically significantly related to workplace burnout levels, which is in line with the results of this study [33]. Based on findings from a systematic review examining interventions for reducing burnout in physicians and nurses, it was revealed that the most commonly employed strategies included enhancements in communication skills, teamwork, participatory programs, and psychological interventions such as yoga, meditation, and mindfulness. These interventions demonstrated potential long-term benefits in mental health outcomes [34].

Early identification of lower resilience may be the key to efficient prevention of negative thoughts and feelings, which can, consequentially, lead to the development of depression and suicidal tendencies. Measuring workplace resilience may also help identify those healthcare workers who are risking leaving their job due to health issues that are a consequence of lower resilience [35]. In this study, significantly higher BRS scale values were seen with participants who had no workplace burnout syndrome. Some of the previous studies showed a negative correlation between resilience and burnout syndrome in nurses [36]. Similarly, another study had similar results and showed a negative association between burnout syndrome and innate resilience [37]. Previous research has shown that resilience interventions combining cognitive behavioral therapy and mindfulness techniques have a positive impact on individual resilience [38].

While this study offers valuable insights into burnout among the sampled population, several limitations should be noted. Firstly, this study was constrained by a relatively small sample size, which may limit the generalizability of the findings in larger populations. Additionally, the unequal distribution between males and females in this sample introduces potential biases and may limit the ability to draw gender-specific conclusions. One more limitation of our research is the absence of a longitudinal perspective, which could provide valuable insights into the development or evolution of burnout in relation to fluctuating work conditions or life stages. It is important to note that our study may be limited by the potential for bias introduced through the reliance on self-reported measures.

## 5. Conclusions

Overtime is a significant factor associated with workplace burnout. This research showed several other factors associated with workplace burnout, including heightened depression, anxiety, and stress, which was somewhat expected considering that mental health issues in healthcare workers show high levels of coexistence. In addition to this, it is important to highlight the effect of early identification of lower resilience, as it can lead to efficient prevention of negative thoughts and feelings.

## Figures and Tables

**Table 1:** Average values, variability, and the distribution of the Copenhagen workplace burnout questionnaire scale and its sub-scales.

Question	Mean	SD	Median	Min	Max	Skewness	Kurtosis
PB1	60.6	20.4	50	0	100	−0.29	−0.07
PB2	58.7	21.6	50	0	100	−0.24	−0.46
PB3	50.0	24.6	50	0	100	−0.21	−0.38
PB4	45.5	26.4	50	0	100	−0.07	−0.71
PB5	51.9	24.4	50	0	100	−0.10	−0.53
PB6	41.4	25.1	50	0	100	0.11	−0.58
WRB1	69.0	23.2	75	0	100	−0.52	0.08
WRB2	62.5	23.3	75	0	100	−0.27	−0.21
WRB3	48.9	24.4	50	0	100	−0.04	−0.26
WRB4	63.0	25.2	75	0	100	−0.23	−0.48
WRB5	49.0	28.0	50	0	100	0.06	−0.74
WRB6	46.7	25.7	50	0	100	0.20	−0.45
WRB7	45.6	28.8	50	0	100	−0.09	−0.97
CRB1	70.8	23.4	75	0	100	−0.44	−0.21
CRB2	54.2	26.3	50	0	100	−0.21	−0.39
CRB3	63.7	25.1	75	0	100	−0.33	−0.34
CRB4	60.5	28.7	75	0	100	−0.40	−0.55
CRB5	54.6	26.2	50	0	100	−0.23	−0.36
CRB6	56.7	28.7	50	0	100	−0.35	−0.55

(PB—personal burnout; WRB—work-related burnout; CRB—client-related burnout; Mean—arithmetic mean; SD—standard deviation; Median—median; Min—minimum value; Max—maximum value).

**Table 2:** The distribution of social–demographic and social–economic characteristics of the participants in relation to the presence of workplace burnout syndrome.

Variables	Has Workplace Burnout Syndrome (*n* = 290)	Does Not Have Workplace Burnout Syndrome (*n* = 201)	*p*-Value
Gender, *n* (%)			
Male	28 (9.7%)	32 (15.9%)	0.037
Female	262 (90.3%)	169 (84.1%)	
Age, am ± SD	46.8 ± 9.7	44.6 ± 11.0	0.021
Age categories, *n* (%)			
20–30	24 (8.3%)	24 (11.9%)	0.029
31–40	45 (15.5%)	55 (27.4%)	
41–50	111 (38.3%)	53 (26.4%)	
51–60	93 (32.1%)	54 (26.9%)	
over 60	17 (5.9%)	15 (7.5%)	
Residence, *n* (%)			
City	251 (86.6%)	179 (89.1%)	0.408
Country	39 (13.4%)	22 (10.9%)	
Marital status, *n* (%)			
Married	85 (29.3%)	73 (36.3%)	0.145
Single	134 (46.2%)	96 (47.8%)	
Divorced	43 (14.8%)	19 (9.5%)	
Widowed	15 (5.2%)	9 (4.5%)	
Other	13 (4.5%)	4 (2.0%)	
Education, *n* (%)			
High School	156 (53.8%)	77 (38.3%)	0.002
College	41 (14.1%)	43 (21.4%)	
Specialist studies/vocational	19 (6.6%)	11 (5.5%)	
BSc/BA, MSc/MA, PhD	74 (25.5%)	70 (34.8%)	
Пoтoмствo, *n* (%)	223 (76.9%)	138 (68.7%)	0.042
Children in family, median (range)	2 (0–5)	1 (0–4)	0.142
Family members, median (range)	4 (1–8)	4 (1–12)	0.549
Homeowner, *n* (%)	209 (72.1%)	139 (69.2%)	0.485
Sole provider, *n* (%)	123 (42.4%)	71 (35.3%)	0.114
Self-assessment of monthly income, *n* (%)			
Very bad	23 (7.9%)	4 (2%)	<0.001
Bad	60 (20.7%)	29 (14.4%)	
Average	165 (5.9%)	122 (60.7%)	
Good	39 (13.4%)	41 (20.4%)	
Very good	3 (1%)	5 (2.5%)	

**Table 3:** The distribution of work environment of the participants in relation to the presence of workplace burnout syndrome.

Variables	Has WBS (*n* = 290)	Does Not Have WBS (*n* = 201)	*p*-Value
Institutions, *n* (%)			
Šekspirova	77 (26.6%)	53 (26.4%)	0.057
Zvečanska	92 (31.7%)	76 (37.8%)	
Sremčica	30 (10.3%)	8 (4%)	
Beograd	91 (31.4%)	64 (31.8%)	
Occupation, *n* (%)			
Healthcare worker	119 (41%)	85 (42.3%)	0.114
Expert worker	83 (28.6%)	71 (35.3%)	
Professional associate	24 (8.3%)	17 (8.5%)	
Associate	64 (22.1%)	28 (13.9%)	
Work experience in the field, median (range)	17 (3–40)	14 (3–41)	0.032
Time spent in current position, median (range)	13.0 (2–40)	11 (2–41)	0.111
Overtime, *n* (%)	106 (36.6%)	48 (23.9%)	0.003
Shifts work, *n* (%)	209 (72.1%)	144 (71.6%)	0.918
Management position, *n* (%)	31 (10.7%)	30 (14.9%)	0.162
Sufficient resources, *n* (%)	151 (52.1%)	148 (73.6%)	<0.001
Spacious and pleasant rooms, *n* (%)	164 (56.6%)	161 (80.1%)	<0.001
Commuting, *n* (%)			
Up to 30 min	83 (28.6%)	66 (32.8%)	0.282
30–60 min	110 (37.9%)	75 (37.3%)	
>60 min	97 (33.4%)	60 (29.9%)	
Means of transport, *n* (%)			
Public	216 (74.5%)	152 (75.6%)	0.958
Car	57 (19.7%)	38 (18.9%)	
Cycling/on foot	17 (5.9%)	11 (5.5%)	

**Table 4:** The distribution of participants’ habits in relation to workplace burnout syndrome presence.

Variables	Has WBS (*n* = 290)	Does Not Have WBS (*n* = 201)	*p*-Value
Nicotine use, *n* (%)	144 (49.7%)	80 (39.8%)	0.031
Number of cigarettes, *n* (%)			
0	146 (50.3%)	121 (60.2%)	0.037
Up to 10	44 (15.2%)	25 (12.4%)	
10–20	76 (26.2%)	42 (20.9%)	
over 20	24 (8.3%)	13 (6.5%)	
Alcohol use, *n* (%)	166 (57.2%)	112 (55.7%)	0.738
Over 5 drinks, *n* (%)			
No	257 (88.6%)	172 (85.6%)	0.340
Once	28 (9.7%)	27 (13.4%)	
At least once per month	5 (1.7%)	2 (1%)	
Sick leave, *n* (%)	119 (41%)	52 (25.9%)	0.001
Sick leave days, median (range)	0 (0–300)	0 (0–120)	<0.001
Self-assessed health, *n* (%)			
Very bad	10 (3.4%)	0 (0%)	<0.001
Bad	48 (16.6%)	5 (2.5%)	
Average	145 (50%)	77 (38.3%)	
Good	74 (25.5%)	98 (48.8%)	
Very good	13 (4.5%)	21 (10.4%)	

**Table 5:** The distribution of mental health symptoms (depression, anxiety, stress, resilience) in relation to WBS presence.

Variables	Has WBS (*n* = 290)	Does Not Have WBS (*n*= 201)	*p*-Value
DASS-21 D, *n* (%)			
Normal	138 (47.6%)	177 (88.1%)	<0.001
Mild	49 (16.9%)	16 (8%)	
Average	53 (18.3%)	7 (3.5%)	
Serious	25 (8.6%)	1 (0.5%)	
Very serious	25 (8.6%)	0 (0%)	
DASS-21 A, *n* (%)			
Normal	99 (34.1%)	161 (80.1%)	<0.001
Mild	20 (6.9%)	14 (7%)	
Average	70 (24.1%)	18 (9%)	
Serious	39 (13.4%)	5 (2.5%)	
Very serious	62 (21.4%)	3 (1.5%)	
DASS-21 S, *n* (%)			
Normal	121 (41.7%)	169 (84.1%)	<0.001
Mild	47 (16.2%)	25 (12.4%)	
Average	54 (18.6%)	2 (1%)	
Serious	48 (16.6%)	5 (2.5%)	
Very serious	20 (6.9%)	0 (0%)	
BRCS, *n* (%)			
Low resilience	109 (37.6%)	36 (17.9%)	<0.001
Average resilience	139 (47.9%)	103 (51.2%)	
High resilience	42 (14.5%)	62 (30.8%)	
BRS average score, am ± sd	2.84 ± 0.60	3.44 ± 0.59	<0.001

**Table 6:** Multivariate logistic regression analysis with WBS as the outcome variable.

Independent Variable	B	*p*	OR	95% Trust Interval	
				Lower Limit	Upper Limit
Gender (F per M)	−0.081	0.838	0.92	0.42	2.01
Age (years)	0.018	0.340	1.02	0.98	1.06
Education level	0.136	0.190	1.15	0.93	1.40
Children	0.206	0.501	1.23	0.68	2.23
Self-assessed monthly income	−0.086	0.616	0.92	0.65	1.29
Work experience (years)	−0.007	0.665	0.99	0.96	1.03
Overtime	0.962	0.001	2.62	1.50	4.56
Sufficient resources	−0.457	0.128	0.63	0.35	1.14
Spacious and pleasant rooms	−0.590	0.063	0.55	0.30	1.03
Self-assessed health level	−0.513	0.004	0.60	0.42	0.85
Degree of BRCS	−0.299	0.107	0.74	0.52	1.07
BRS average	−1.291	<0.001	0.28	0.17	0.44
DASS21 D heightened depression	0.735	0.029	2.09	1.08	4.04
DASS21 A heightened anxiety	0.867	0.003	2.38	1.34	4.21
DASS21 S heightened stress	0.732	0.022	2.08	1.11	3.89

## Data Availability

Data available on request due to restrictions (e.g., privacy, legal or ethical reasons).
